# Quinolinic acid injection in mouse medial prefrontal cortex affects reversal learning abilities, cortical connectivity and hippocampal synaptic plasticity

**DOI:** 10.1038/srep36489

**Published:** 2016-11-07

**Authors:** Amira Latif-Hernandez, Disha Shah, Tariq Ahmed, Adrian C. Lo, Zsuzsanna Callaerts-Vegh, Annemie Van der Linden, Detlef Balschun, Rudi D’Hooge

**Affiliations:** 1 Laboratory of Biological Psychology, Brain and Cognition, KU Leuven, Tiensestraat 102, 3000 Leuven, Belgium; 2 Bio-Imaging Lab, University of Antwerp, Universiteitsplein 1, 2610 Wilrijk, Antwerp, Belgium; 3 Laboratory for Molecular Neurobiology, VIB center for the biology of disease, KU Leuven, Herestraat 49, 3000 Leuven, Belgium; 4 Departement of Fundamental Neurosciences, University of Lausanne, Rue du Bugnon 7-9, 1005 Lausanne, Switzerland

## Abstract

Intracerebral injection of the excitotoxic, endogenous tryptophan metabolite, quinolinic acid (QA), constitutes a chemical model of neurodegenerative brain disease. Complementary techniques were combined to examine the consequences of QA injection into medial prefrontal cortex (mPFC) of C57BL6 mice. In accordance with the NMDAR-mediated synapto- and neurotoxic action of QA, we found an initial increase in excitability and an augmentation of hippocampal long-term potentiation, converting within two weeks into a reduction and impairment, respectively, of these processes. QA-induced mPFC excitotoxicity impaired behavioral flexibility in a reversal variant of the hidden-platform Morris water maze (MWM), whereas regular, extended MWM training was unaffected. QA-induced mPFC damage specifically affected the spatial-cognitive strategies that mice use to locate the platform during reversal learning. These behavioral and cognitive defects coincided with changes in cortical functional connectivity (FC) and hippocampal neuroplasticity. FC between various cortical regions was assessed by resting-state fMRI (rsfMRI) methodology, and mice that had received QA injection into mPFC showed increased FC between various cortical regions. mPFC and hippocampus (HC) are anatomically as well as functionally linked as part of a cortical network that controls higher-order cognitive functions. Together, these observations demonstrate the central functional importance of rodent mPFC as well as the validity of QA-induced mPFC damage as a preclinical rodent model of the early stages of neurodegeneration.

Intracerebral injection of the excitotoxic, endogenous tryptophan metabolite, quinolinic acid (QA), has been used as a chemical model of neurodegeneration in rodents for more than 30 years already^1^. The biochemical and metabolic changes induced by intracerebral QA injection putatively resemble or even reproduce many aspects of the complex pathophysiology of human neurodegenerative disorders such as Alzheimer’s disease^2^. The pathophysiology of such brain disorders has been suggested to include excitotoxic phenomena^3^, which commence in telencephalic areas such as hippocampus (HC) and prefrontal cortex (PFC), and eventually affect synapses and cells across the brain, as well as the connectivity between brain areas^4^,^5^. Examination of the effects of QA-induced excitotoxic PFC lesions may therefore not merely help to understand the importance of PFC-controlled cortical functions and interactions, but also provide crucial insights into the early phases of neurodegeneration.

It has been well documented that the ability to adjust behavior flexibly in response to changing environmental demands, a central feature of higher-order cognition, is affected earliest in Alzheimer’s disease patients^6^. Reversal learning, defined as the modulation or adaptation of learned responses, is the most reliable behavioral paradigm to model cognitive flexibility in laboratory rodents^7^,^8^,^9^. Reversal learning has been examined in various cognitive tasks, including the leading procedure to study complex learning and memory in rats and mice, namely the Morris water maze (MWM), spatial navigation task^10^,^11^. During MWM reversal, spatial information is updated by subsequent experience, and performance depends on inhibition and adaptation of previously learned strategies that animals use to search for the hidden platform^12^,^13^. Notably, MWM reversal was affected by experimentally induced synaptic defects in telencephalic areas that express vesicular glutamate transporter 1, which includes HC, medial PFC (mPFC), and associated (sub)cortical regions^12^.

MWM learning essentially relies on HC synaptic plasticity, consistent with the established role of this brain area in spatial navigation and contextual information storage, whereas mPFC appears to play a distinct, albeit somewhat less straightforward role in MWM learning^14^,^15^,^16^,^17^,^18^. The precise role of mPFC in spatial learning is still being debated, but mPFC and HC appear to be anatomically as well as functionally linked as part of a cortical network that controls higher-order brain processes in primates and other mammals^19^,^20^,^21^. Indeed, prelimbic (PL) and infralimbic (IL) parts of mPFC receive direct input from CA1 region of HC^22^,^23^, and there also exist indirect connections between PL/IL and HC via *nucleus reuniens* (NRe) in midline thalamus^24^,^25^. Lesions in mPFC hardly affect spatial memory functions proper, but rather impair the flexibility of MWM performance^26^,^27^,^28^,^29^,^30^,^31^,^32^, which is consistent with a more general function of mPFC in executive control, decision making, and most notably, cognitive flexibility^33^,^34^,^35^.

In the present study, we injected QA into mPFC (including PL and IL) of laboratory mice to examine the functional consequences of excitotoxic damage to this brain region and learn about the (executive) role of rodent mPFC. However, we also wanted to assess the validity of QA injections into mPFC as a preclinical rodent model of early neurodegenerative pathology. Therefore, we first studied the effect of QA injections on behavioral flexibility using a reversal learning version of the hidden-platform MWM. Several studies indicated that mPFC plays some, rather difficult to define role in MWM learning^14^,^15^,^16^,^17^,^18^. We therefore examined whether QA injections would affect the cognitive strategies that mice use to locate the hidden platform during reversal learning. Indeed, the adaptive use of cognitively demanding spatial search strategies has been shown to be impaired in some genetic models of neurodegenerative brain disease^36^. We further hypothesized that the behavioral and cognitive effects of mPFC lesions might actually be attributed to changes in functional connectivity (FC) between various cortical regions. We therefore included resting-state functional MRI (rsfMRI) methodology, which previously allowed us to describe FC defects in another mouse model of degenerative brain disease^37^. Finally, we examined the acute and delayed effects of QA injections into mPFC on hippocampal synaptic plasticity, which is considered to be the cellular substrate of spatial learning abilities^38^,^39^.

## Material and Methods

### Animals and experimental groups

For all experiments, 8-week-old female C57Bl/6J mice were purchased from Elevage Janvier (Le-Genest Saint-Isle, France), and handled for a week before experiments started. Before surgery, mice were randomly assigned to sham-treated and PL/IL lesion groups (see Table 1). The study as a whole included three experiments on three different batches of mice that differed in terms of toxin injection time and MWM protocol. The first experiment (EXP1) included mice that were only used for hippocampal long-term potentiation (LTP) recordings following acute QA injection into PL/IL. The more extensive EXP2 included groups of lesioned and sham-treated mice that were used in MWM acquisition and reversal, hippocampal LTP, and rsfMRI procedures. EXP3 included mice that were used for extended MWM acquisition and subsequent LTP recording. Lesion site was histologically confirmed at the end of each experiment, and mice that failed to show PL/IL lesions were excluded from all analyses (3 mice died during the experiments). The number of mice indicated in the table thus refers to the number after lesion site confirmation. Two mice were discarded from EXP1 due to technical problems during LTP experiments. Mice were group-housed according to treatment condition to prevent possibly harmful interactions between sham and lesioned animals. All animals were kept in standard animal cages under conventional laboratory conditions with *ad libitum* access to food and water (lights on: 8:00–20:00 h; lights off: 20:00–8:00 h; 22 °C). Experiments were conducted during the light phase of the animals’ activity cycle. All experimental procedures were reviewed and approved by the *KU Leuven animal ethics committee* (P073/2013) in accordance with European Community Council Directive (86/609/EEC) guidelines and regulations.

### Intracerebral QA injection

QA injections into mPFC were either performed after two weeks of MWM acquisition training, (i.e., before reversal) in EXP2, before extended training on the same platform position in EXP3 or 24 h prior to LTP recordings (EXP1). Animals were anesthetized with Nembutal (5 mg/kg) and chloral hydrate (25 mg/kg, i.p., 7–9 minutes after Nembutal injection), this combination prevented QA-induced seizures during postoperative recovery. The scalp was locally anesthetized with xylocaine, and respiratory depression was alleviated by oxygen application. The mice were placed in a stereotactic frame and, after incision of the skin, two small holes were drilled in the skull at the appropriate locations. A 30 nmol dose of quinolinic acid (QA), dissolved in 0.1 M phosphate-buffered saline (pH 7.4), was bilaterally injected (left hemisphere first; injection volume 0.1 μl). The following coordinates according to Franklin and Paxinos were used to target PL and IL cortices bilaterally: anteroposterior (AP), mediolateral (ML), and dorsoventral (DV, from brain surface), relative to Bregma, AP + 2 mm, ML ± 0.25 mm, DV 1.8 mm. PL and IL are relatively small regions that are situated adjacently in mouse mPFC^40^, which makes it difficult to target them independently by stereotactic injection.

Each injection lasted 4 min with the needle left *in situ* for another 2 mins, then raised 0.5 mm and left another minute, before being slowly withdrawn. After surgery, the skin was closed with silk sutures, and the animal was put on a warm pad to prevent hypothermia. Sham-operated animals underwent the same manipulations including anesthesia and scalp incision, except that vehicle was given instead of neurotoxin solution. Peri-surgical analgesia was provided for 48 h by paracetamol (1.6 mg/ml in drinking water). Animals were allowed to recover for 7–8 days before behavioral testing or rsfMRI scanning. In EXP2, half of the lesioned animals were assigned to MWM reversal (n = 13 after confirmation of lesion site), and the other half to neuroimaging (n = 12 after confirmation of lesion site). The same was done with the sham group (n = 10 in both cases).

### Spatial and reversal learning in the Morris water maze

Mice were first trained during 10 daily acquisition sessions to find the hidden platform (15 cm diameter) in a typical hidden-platform Morris water maze (MWM, circular pool, 150 cm diameter) filled with opaque water (non-toxic white paint, temperature 26 ± 1 °C) as previously described^17^,^41^,^42^. Each daily session consisted of 4 swimming trials (15 min intertrial interval), starting randomly from each of 4 starting positions. Swimming tracks were recorded using video hardware and Ethovision software (Noldus, The Netherlands). Mice that failed to find the platform within 2 min were guided to it and remained there for 15 s before being returned to their cages. To assess retention memory, interspersed probe trials (100 s, platform removed) were conducted after 5 and 10 acquisition sessions. Heat maps were generated to represent the time spent in different parts of the pool during these probe trials. After establishing robust spatial preference for the platform location (10 daily sessions), either reversal trials were performed during which the platform was placed in a different location (EXP2), or training was continued for another week on the same position (EXP3). Both MWM experiments thus lasted 15 days. In addition to swimming track and escape latency recording, search strategies were analyzed during reversal learning. We used an approach described previously to classify the search strategies^43^. Briefly, each track was classified to a particular strategy using binary support vector machine (SVM) classifiers^44^ and a previously described 9-category scoring system (see Table 2).

The manner in which mice search for the hidden platform can be categorized into three main strategies, which can be further subdivided into several subcategories. Firstly, spatial strategies that rely on distal cues, can be further subcategorized as “spatial direct” (i.e., animal swims towards the platform in a straight line), “spatial indirect” (i.e., animal swims in a more circumlocutory manner towards the platform) and “focal correct” (i.e., animal searches for the platform in the correct quadrant). Secondly, non-spatial strategies do not rely on spatial cues, but are still systematic and may concentrate on a specific part of the pool. These can be subcategorized as “scanning” (i.e., confined largely to the center of the pool), “focal incorrect” (i.e., confined to a non-target quadrant) and “random” (i.e., with no specific preference to one particular part of the pool). Finally, repetitive strategies comprise repetitive concentric-circle searches, which can be further categorized as “peripheral looping” (i.e., animal swims in concentric circles along the pool border or wall hugging), “circling” (i.e., animal swims in tight concentric circles that resemble tail-chasing) and “chaining” (i.e., animal swims in concentric circles at a fixed distance from the wall). Perseveration is considered a separate class of search strategy based on previous experience. In this case, the mouse confines its search to the previously rewarded quadrant.

### Resting-state functional MRI procedure

Prior to the scanning session, mice were anesthetized with 2% isoflurane (IsoFlo, Abbott, Illinois, USA), in a gas mixture of 70% N_2_ and 30% O_2_. The physiological status of all animals was monitored throughout the procedure using a pressure-sensitive pad (MR-compatible Small Animal Monitoring and Gating System, SA Instruments) to monitor breathing rate, and a rectal thermistor with feedback-controlled warm air circuitry (MR-compatible Small Animal Heating System, SA Instruments) to maintain body temperature at 37.0 ± 0.5 °C. During scanning, a combination of medetomidine (0.3 mg/kg; Domitor, Pfizer, Karlsruhe, Germany) and isoflurane (0.4%) was used. After scanning, the effects of medetomidine were counteracted by 0.1 mg/kg atipamezole (Antisedan, Pfizer). Resting-state imaging (rsfMRI) was performed on a 9.4T Biospec MRI system (Bruker BioSpin, Germany) with Paravision 5.1 software (www.bruker.com). Images were acquired using a standard Bruker cross coil set-up with a quadrature volume and surface coil for mice. Three orthogonal multi-slice Turbo RARE T2-weighted images were acquired to allow uniform slice positioning (repetition time 2000 ms, echo time 15 ms, 16 slices of 0.4 mm). Field maps were acquired for each animal to assess field homogeneity, followed by local shimming, which corrects for inhomogeneity in a rectangular brain VOI. Resting-state signals were measured during a T2*-weighted single shot EPI sequence (repetition time 2000 ms, echo time 15 ms, 16 slices of 0.4 mm, 150 repetitions). The field-of-view was 20 × 20 mm^2^ and the matrix size 128 × 64 mm^2^, resulting in voxel dimensions of 0.16 × 0.31 mm^2^.

### Imaging data analysis

Pre-processing of rsfMRI recordings, including realignment, normalization and smoothing, used SPM8 software (Statistical Parametric Mapping, http://www.fil.ion.ucl.ac.uk). Images within each session were first realigned to the first image. This was done using a least-squares approach and a 6-parameter (rigid body) spatial transformation. All datasets were consequently normalized to a defined EPI template. This normalization step consisted of a global 12-parameter affine transformation followed by estimation of nonlinear deformation. Eventual in-plane smoothing was done with a Gaussian kernel of twice the voxel size. rsfMRI data were filtered between 0.01 and 0.1 Hz using the REST toolbox (REST1.7, http://resting-fmri.sourceforge.net). Motion parameters, resulting from the realignment, were included as covariates to correct for movements that occurred during scanning.

Analysis consisted of two major steps. First, seed-based analysis was performed using right prefrontal cortex as seed region. Using the REST toolbox, individual z-transformed FC maps were obtained for all animals (i.e., sham and lesioned mice). These individual z-transformed FC maps were loaded in SPM8, and mean zFC maps were computed per group. A statistical difference map was obtained showing all voxels that were significantly different between the two groups (i.e., voxels that show differential FC with the right prefrontal cortex between sham and lesioned animals). This difference map was shown as an overlay on the EPI template.

Next, the REST toolbox was used to compute z-transformed FC matrices for each subject using cortical regions that had shown different FC between the groups during seed-based analysis (i.e., prefrontal cortex, motor cortex, cingulate and retrosplenial cortex, somatosensory cortex, and hippocampal CA1 region). The time course of BOLD signals were extracted for each of these regions, and z-transformed correlation coefficients between time traces of each region pair were calculated and represented in a correlation matrix. Mean zFC matrices were calculated for each experimental group using MATLAB (MATLAB R2013a, MathWorks Inc. Natick, MA, USA). Additionally, these matrices were used to calculate FC strength for each cortical region (i.e., mean strength of the correlation between a specific region and all other regions in the matrix).

### Hippocampal LTP recordings

After completion of reversal sessions (EXP2) or extended acquisition training (EXP3), mice were killed by cervical dislocation, and HC was rapidly dissected in ice-cold (4 °C) artificial cerebrospinal fluid (ACSF), saturated with carbogen (95% O_2_/5% CO_2_). ACSF consisted of (in mM): 124 NaCl, 4.9 KCl, 24.6 NaHCO_3_, 1.20, KH_2_PO_4_, 2.0 CaCl_2_, 2.0 MgSO_4_, 10.0 glucose, pH 7.4. Transverse hippocampal slices (400 μm thick) were prepared from the dorsal area of the right HC with a tissue chopper and placed into a submerged-type chamber, where they were kept at 32 °C and continuously perfused with ACSF at a flow-rate of 2.4 ml/min. After 90 min incubation, one slice was selected and a tungsten stimulation electrode was placed in the CA1 *stratum radiatum*. For recording of field excitatory postsynaptic potentials (fEPSPs), a glass electrode (filled with ACSF, 3–7 MΩ) was placed in *stratum radiatum* opposite the stimulation electrode. The descending slope of the fEPSP was measured and used as the primary response variable in all recordings. After input/output relationships were established, stimulation strength was adjusted to elicit a fEPSP slope at 35% maximal value, which was maintained throughout the experiment. During baseline recording, 3 single stimuli (0.1 ms pulse width; 10 s interval) were measured every 5 min and averaged during 40 min fEPSP intervals. A single theta burst stimulation (TBS) was employed for LTP induction, which consisted of 10 burst of four 100 Hz stimuli, separated by 200 ms (double pulse width), and followed by responses evoked at 1, 4 and 7 min.

### Histological post-mortem confirmation of injection site

**Figure 1:**
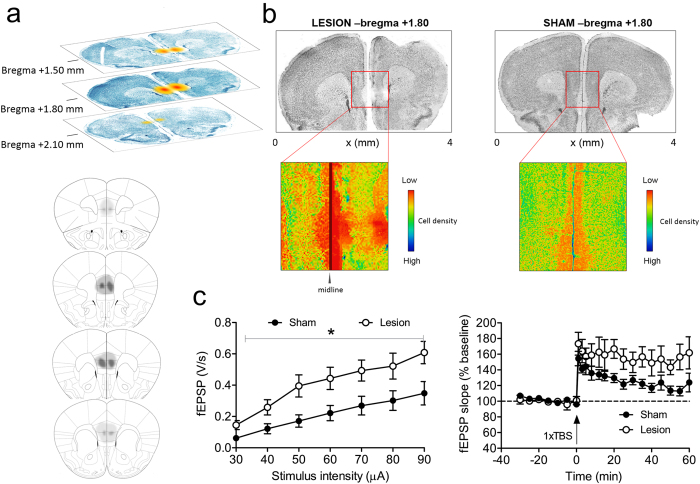
Acute hippocampal hyperexcitability and eventual excitotoxic mPFC lesions resulting from QA injections into mPFC. (**a**) The upper part of this figure panel illustrates lesion size at different coronal slice levels in a representative animal. The schematic diagram in the bottom part delineates the largest (dark grey) and smallest (light grey) extent of QA-induced damage across the PL and IL areas. (**b**) Photomicrographs show representative mPFC lesions, 1.80 mm anterior to *bregma*. We show a representative section from a lesioned mouse (on the left) and a sham animal (on the right). Enlarged and graphically enhanced images of the lesion area, shown underneath, illustrate the areas with decreased cell density, clearly delineating the damaged areas. (**c**) Acute effects of mPFC QA injection (24 h before recording) on HC electrophysiology. The subfigure on the left demonstrates that QA injection into mPFC acutely increases synaptic transmission in hippocampal CA1 region. Input/Output (I/O) curves reveal a significant increase in evoked fEPSPs after QA injection. Also, LTP is enhanced by QA injection in mPFC (shown on the right). Data are presented as means ± SEM.

After the last testing day, animals were killed by cervical dislocation, followed by decapitation. Brains were removed and postfixed in 4% formaldehyde solution for histological confirmation of the injection site. Coronal sections (30 μm thick) were cut on a vibratome, placed on gelatin-coated slides, air-dried and Nissl-stained with 1% cresylviolet (Fluka Chemical, Sigma-Aldrich) according to standard procedures. These sections were examined under a Leica DM RBE microscope (Leica, Leitz Instruments, Heidelberg, Germany) for histological verification of the lesions. Light microscopic examination of Nissl-stained brain sections revealed that our QA injections typically produced prefrontal lesions that extended from ventral PL to dorsal IL area. The damaged area was highlighted and demarcated using *Gwyddion* free software. The software converted a selected area of the photomicrographs into a (linear) multicolor scale to increase contrast. A threshold contrast value was defined to estimate the fraction of pixels in the damaged brain area. The volume of the lesion was approximated by an ellipsoid (shown in the inset of Fig. 1a), using 3 brain slices (bregma + 1.50, +1.80 and +2.10 mm; 30 μm thickness) to extract its radius values. Animals with lesions that extended to anterior cingulate cortex or *nucleus accumbens*, and/or showed an otherwise asymmetrical pattern of damage, were discarded from further analysis.

### Statistics

Data are presented as means ± SEM. Significance of differences between mean values were determined using t-test or 2-way repeated measures analysis of variance (RM-ANOVA) with Fisher LSD tests for *post hoc* comparison; p < 0.05 was considered significant. Statistical differences between input/output and LTP curves were analyzed by ANOVA with repeated measures (RM-ANOVA) using SPSS 19 (IBM Corp., Armonk, N.Y., USA). Non-linear regression analysis of LTP data was done with GraphPad Prism 5. This data was analyzed and graphs were plotted using Sigmastat 3.1 and Sigmaplot 9.0, respectively (Point Richmond, CA, USA). Statistical analyses of rsfMRI data and FC maps used SPM8. Two sample t-test was used to compare right prefrontal cortex FC between groups (uncorrected, p < 0.001, voxel threshold of 10 voxels). Two sample t-tests (with Bonferroni correction for multiple comparisons, p < 0.05) compared FC strength differences using SPSS software (http://www-01.ibm.com/software/be/analytics/spss/).

## Results

### Acute excitatory effects of QA injection and QA-induced mPFC lesions

In EXP1, we examined whether acute intra-mPFC injection of QA influenced CA1 electrophysiology. We injected QA into mPFC (n = 6 QA animals, n = 9 shams), and examined CA1 synaptic transmission and plasticity 24 h later. Slices from QA-injected mice showed significantly higher I/O responses than sham controls (F_1,16_ = 6.5, p = 0.02, RM-ANOVA; see Fig. 1c, left panel). Accordingly, QA-injected mice expressed a more robust LTP as compared to the decremental potentiation in sham animals. This led to a statistically significant difference during the second half of the recording period (F_1,11_ = 5.7, P = 0.036, RM-ANOVA; see Fig. 1c, right panel) and is also supported by the results of a non-linear regression with a one-phase exponential decay [Y = (Y0-Plateau)*exp(-K*X) + Plateau; x: time, K: rate constant, Y starts at Y0 and decays down to plateau]. The regression yielded for the plateau of potentiation values of 117 ± 10% for sham mice, and 153 ± 15% for lesioned animals. These results overall suggest that QA injections into mPFC acutely boosted neuronal excitation and LTP in CA1. The brains of all injected mice were examined after completion of behavioral experiments. Coronal brain sections from representative animals in Fig. 1b illustrate the extent of bilateral lesions in mPFC of shams (right panel) and lesioned animals (left panel). The schematic diagrams in Fig. 1a outline the largest and smallest extent of the lesions in all QA-injected animals. The typical bilateral ellipsoid lesion volume was approximately 2.45 mm^3^, including both IL and PL areas. Four mice were excluded from the analyses because of lesion inaccuracies, according to predefined criteria (see methods).

### mPFC lesions impair MWM reversal learning

**Figure 2:**
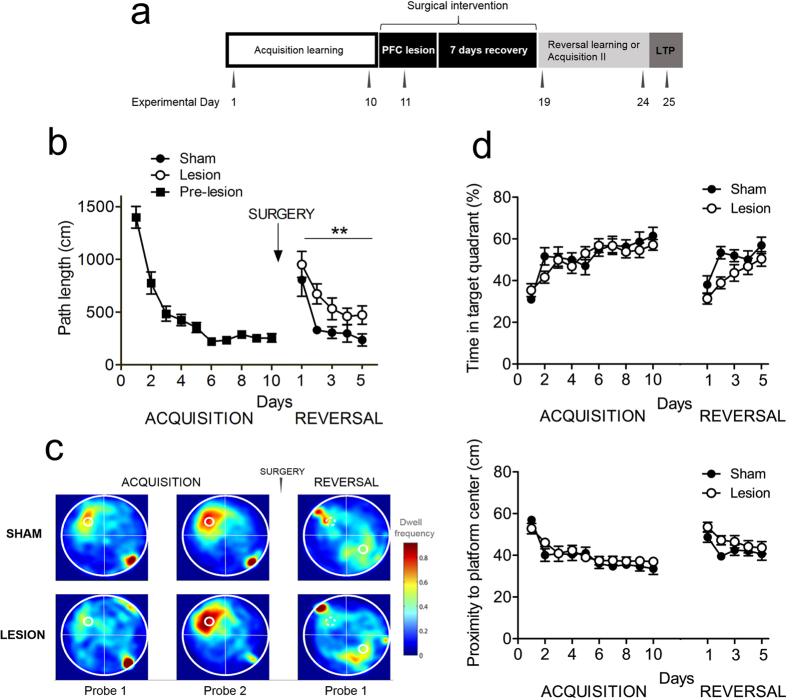
QA-induced mPFC damage impairs reversal learning in the Morris water maze. (**a**) Schematic of the experimental design. (**b**) Mice with mPFC damage (open circles) performed worse than sham-operated mice (closed circles) in acquiring new information during the reversal phase. Number of days during each phase (acquisition and reversal) is presented on the horizontal axis, average distance travelled is presented on the vertical axis. Arrow indicates the time point of the surgical manipulation. Data are expressed as means ± SEM. (**c**) Heat plots of search intensity during probe trials conducted on day 6 and 11 of acquisition training (first 2 panels from the left); and day 6 of reversal training (right panel). High dwell time across the MWM pool area is indicated by colors close to red, whereas colors close to blue indicate lower dwell time. Lesioned mice (bottom panel) spent more time searching in the previous platform quadrant than the new location, compared to sham animals (upper panel). The small hot spot in the right lower quadrant indicates that animals remain a few seconds at the place of release (orienting), before actually starting to navigate. (**d**) Time in target quadrant (upper panel) and proximity to platform (bottom panel) measurements illustrate slight but significant performance defects in lesioned mice during the reversal phase. See text for statistical analysis.

During pre-surgery acquisition training of EXP2 (Fig. 2b), all mice reached a high level of task proficiency already after a couple of days of training. We chose path length as a performance measure, because this variable is most likely to represent the cognitive aspect of MWM learning and compared to latency, is less likely affected by motor performance. However after surgery, RM-ANOVA revealed a highly significant main effect of treatment group on path length during reversal trials (F_1,21_ = 8.7, p = 0.007), which demonstrated that MWM reversal was markedly impaired in mice with mPFC lesions. However, the main effect of trial block on path length indicated that even the lesioned group retained some learning ability (F_1,23_ = 12.8, p < 0.0001). Day 2 of reversal learning shows the strongest difference in path length between lesioned and sham animals (corrected t_18_ = 3.56, p = 0.002). Notably, swimming velocity was not different between the groups indicating that mPFC lesions did not affect swimming ability as such (F_1,23_ = 1.3, p = 0.26).

Probe trials were interspersed to assess spatial reference memory during the acquisition and reversal phases (Fig. 2c). Heat maps of probes conducted during acquisition and reversal training illustrated that (1) all mice developed a focal search pattern after two weeks of acquisition training (two focal dwell spots in the lower right quadrant in Fig. 2c indicate the first seconds that the animals remained in the opposite quadrant where they were released) (2) this focal search pattern reversed readily in sham mice, whereas mPFC-lesioned animals displayed somewhat more diffuse search patterns after reversal training (again, focal dwell spots, this time in the upper left quadrant, represent the first seconds of the probe trial after the mice were released in the quadrant opposite to the target area).

During acquisition training, we did not find any group differences for time in target quadrant (TQ; Fig. 2d, upper panel) or proximity to platform center (Fig. 2d, bottom panel). In both analyses, we did find a significant main effect of testing day (time in TQ: F_9,230_ = 9.5, p < 0.001; proximity: F_9,230_ = 13.3, p < 0.001), indicating that all mice acquired the task equally efficient. During reversal learning, we found, both for time in TQ and proximity to the platform center, a significant main effect of group (TQ: F_1,110_ = 11.7, p < 0.001; proximity: F_1,110_ = 7.9, p < 0.01) and testing day (TQ: F_4,110_ = 7.8, p < 0.001; proximity: F_4,110_ = 4.0, p < 0.01), but no interaction effect for group by day. This indicates that for these two performance parameters sham mice performed consistently better during reversal training than lesioned mice.

### mPFC lesions affect the use of spatial strategies during reversal learning

**Figure 3:**
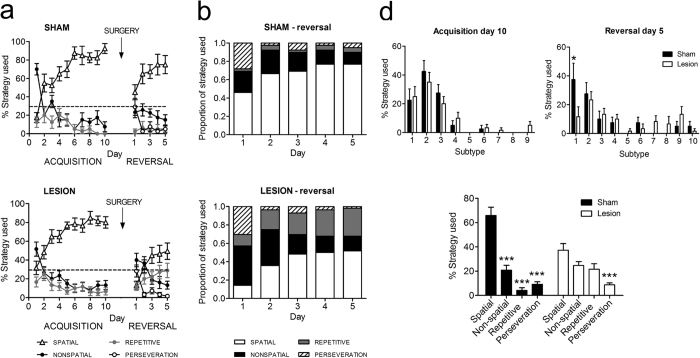
QA-induced mPFC damage affects the use of cognitively demanding spatial search strategies. (**a**) Both sham (upper panel) and QA-injected animals (bottom panel) adopted a prominent use of spatial strategies during the acquisition phase of MWM learning (i.e., before the surgical procedure, white triangles). After surgery, sham mice quickly return to their use of spatial strategies (non-spatial and repetitive strategies as well as perseveration occur well below chance level). In contrast, QA-injected animals never regain their use of spatial strategies, and rather continue to rely on a mixture of other types of search strategies. Data are presented as means ± SEM. (**b**) Increase in the proportional use of different search strategies across 5 days of reversal learning (after surgery) in the sham-treated group. White bars illustrate the increasing use of spatial strategies, reaching a maximum on days 4 and 5. (**c**) Defects in spatial strategy use in QA-injected animals. These mice reached their maximal, more limited use of spatial strategies already on day 3, and continued to rely substantially on repetitive strategies. (**d**) Marked differences in the use of strategy subtypes between control and lesioned mice on the last reversal day. The figure compares between their last day of acquisition (upper left) and reversal (upper right). Number in the x axis corresponds to the different strategy subtypes as listed in Table 2 with (1) spatial direct; (2) spatial indirect; (3) focal correct; (4) scanning; (5) random; (6) focal incorrect; (7) chaining; (8) peripheral looping; (9) circling; (10) perseveration, only possible on reversal days. The bottom panel shows that sham animals almost exclusively used spatial strategies (spatial v. non-spatial: p < 0.001; spatial v. repetitive: p < 0.001; spatial v. perseveration: p < 0.001), whereas lesioned animals do not have a preference for any particular search strategy. However, perseveration was very low in both groups (spatial v. perseveration: p < 0.001).

Typically, mice initially used non-spatial strategies to locate the hidden platform, but as training proceeded and they acquired information about the spatial location of the platform, they increasingly used more cognitively demanding strategies that are based on spatial cue configuration (Fig. 3a). Not surprisingly, all mice were equally efficient, before surgery, in this gradual deployment of spatial search strategies. After surgery and during reversal training, only sham animals were able to continue and adapt their use of spatial strategies (Fig. 3b). We observed significant strategy by day (F_12,276_ = 6.5, p < 0.001) and strategy by group effects (F_3,69_ = 8.6, p < 0.001). Lesioned mice largely failed to deploy spatial strategies during the initial phase of reversal learning, and rather continued to rely on a mixture of strategies. Post-hoc comparison of overall strategies use during MWM reversal learning demonstrated that lesioned mice deploy significantly less spatial strategies (t_23_ = 3.19, p < 0.01), and significantly more repetitive strategies (t_23_ = 3.07, p < 0.01), compared to control mice.

We have separately analyzed the different search strategies for the final acquisition and reversal day. These analyses show that on the final acquisition day (Fig. 3d, left upper panel), sham and to-be-lesioned mice did not show differences in strategy use. On the last reversal day (Fig. 3d, right upper panel), however, we found a significant main effect of strategy (F_9,230_ = 5.7, p < 0.001) and a significant interaction effect of strategy by group (F_9,230_ = 2.1, p < 0.05). Post-hoc comparisons showed that sham mice used significantly more often “spatial direct” strategies than lesioned mice (p < 0.05; Fig. 3d, bottom panel). We found that there was a significant main effect of strategy (F_3,92_ = 36.9, p < 0.001) as well as a significant interaction effect of strategy by group (F_3,92_ = 10.7, p < 0.001). Post-hoc pairwise comparison shows that sham animals almost exclusively used spatial strategies (spatial v. non-spatial: p < 0.001; spatial v. repetitive: p < 0.001; spatial v. perseveration: p < 0.001), whereas lesioned animals do not have a preference for any particular search strategy. However, perseveration was very low in both groups (spatial v. perseveration: p < 0.001).

### mPFC lesions alter cortical connectivity

**Figure 4:**
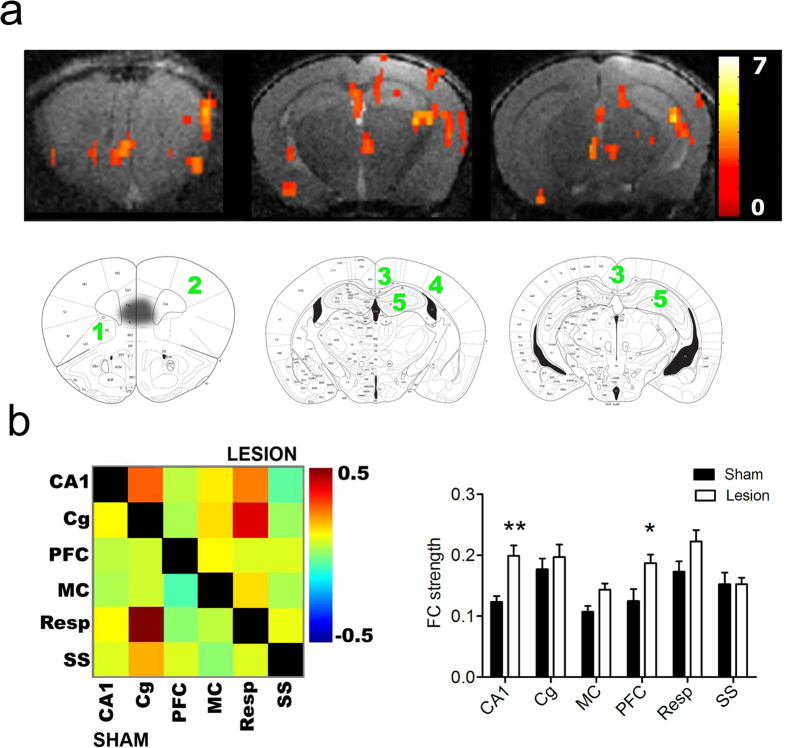
QA injections enhance functional connectivity (FC) between different cortical areas of the mouse brain. (**a**) Seed-based analysis of the right prefrontal cortex, FC matrices and FC strength in 3 slices of the statistical difference map between sham (n = 10) and QA-injected mice (n = 12; p < 0.001, uncorrected, voxel threshold of 10 voxels; lesion >sham). The color scale on the right indicates the *T* value (i.e., the magnitude of the difference between both conditions). The regions that showed higher FC in the QA-injected group are indicated on corresponding slices of the Franklin and Paxinos anatomical mouse brain atlas with (1) prefrontal cortex, (2) motor cortex, (3) cingulate/retrosplenial cortex, (4) somatosensory cortex and (5) CA1 region of hippocampus. Black spot on first brain slice on the left indicates the lesion site. (**b**) The panel on the left side shows mean zFC matrix of the sham (below the diagonal) and QA-injected group (above the diagonal). The panel on the right side shows a graph with mean FC ± standard error for each brain region in sham and lesion groups. **p < 0.01. The color scale represents the strength of the functional correlation between pairs of brain regions. Abbreviations: CA1 = CA1 region of the hippocampus, Cg = cingulate cortex, PFC = prefrontal cortex, MC = motor cortex, Resp = retrosplenial cortex, SS = somatosensory cortex.

Some of the QA-injected mice of EXP2 were used for rsfMRI recordings. Seed-based analysis of PFC showed increased FC in the lesioned animals compared to sham mice (Fig. 4a) within PFC, motor cortex, cingulate/retrosplenial cortex, somatosensory cortex and CA1 region of HC (uncorrected, p < 0.001). None of the brain regions were significantly increased in sham animals. These regions were then included to calculate a FC matrix for each group (Fig. 4b, panel on the left side) which confirmed increased FC in the lesion group (above the diagonal). A comparison of FC strength (Fig. 4b, right panel) shows that PFC (p = 0.008, Bonferroni correction for multiple comparisons) and CA1 region of HC (p = 0.001, Bonferroni correction for multiple comparisons) showed marked FC strength increase in the lesion group.

### mPFC lesions affect CA1 synaptic plasticity after MWM reversal

**Figure 5:**
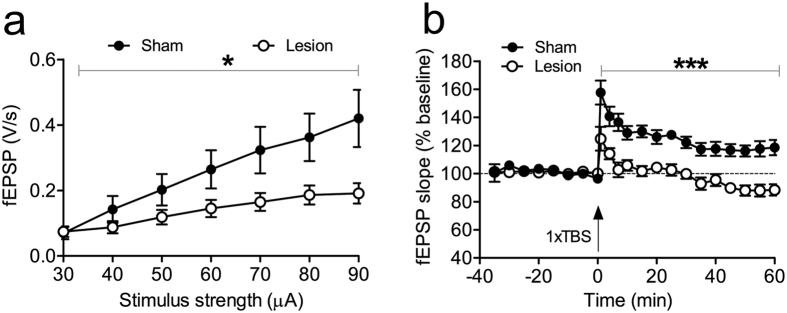
QA injections into mPFC reduce basal synaptic transmission and LTP at Schaffer collateral-CA1 synapses in hippocampal slices after reversal training. (**a**) I/O curves of sham (closed circles) and QA-injected animals (open circles) demonstrate significantly decreased CA1 evoked responses in QA-injected mice. (**b**) LTP was significantly impaired in the CA1-region of QA-injected mice. LTP in the sham group was maintained until the end of recording, whereas TBS induced only a short-lasting potentiation in the QA-injected group. Data are presented as mean ± SEM.

After completion of reversal learning in the MWM (EXP2), we examined basal synaptic transmission and long-term potentiation (LTP) in the hippocampal CA1 region. LTP is widely accepted as a cellular substrate of spatial learning and memory^38^,^39^. Input/output (I/O) curves of fEPSPs at stimulation intensities from 30 to 90 μA revealed that basal CA1 excitability was dramatically reduced in the lesioned animals (F_1,16_ = 4.9, p = 0.04, RM-ANOVA; Fig. 5a). The I/O differences between the groups augmented with increasing stimulation intensity, reaching statistical significance from 60 to 90 μA. TBS induced robust and stable LTP in slices from control mice (i.e., 158 ± 8% of baseline at 1 min post-TBS, 124 ± 5% at 70 min post-TBS; Fig. 5b), but completely failed to induce LTP in brain slices from mPFC-lesioned mice as demonstrated by significantly reduced LTP magnitude compared to sham mice (F_1,18_ = 53.9, p < 0.0001, RM-ANOVA). In slices from lesioned mice, TBS did evoke an initial rise in synaptic strength (128 ± 9% at 1 min post-TBS), but the potentiation was not sustained and recordings returned to baseline level at about 4 min post-TBS.

A comparison of the I/O curves and LTP, registered 24 h and two weeks after QA lesions (i.e., after reversal learning), revealed a marked decrease in basic excitability (F_1,18_ = 29.8, p < 0.0001) and LTP during this period. The latter was augmented 24 h after QA injection, but completely absent after reversal learning two weeks later, resulting in a highly significant difference (F_1,15_ = 29.7, p < 0.0001; compare between Figs 1a and 5a, and between Figs 1c and 5b).

### mPFC lesions do not affect continued MWM performance

**Figure 6:**
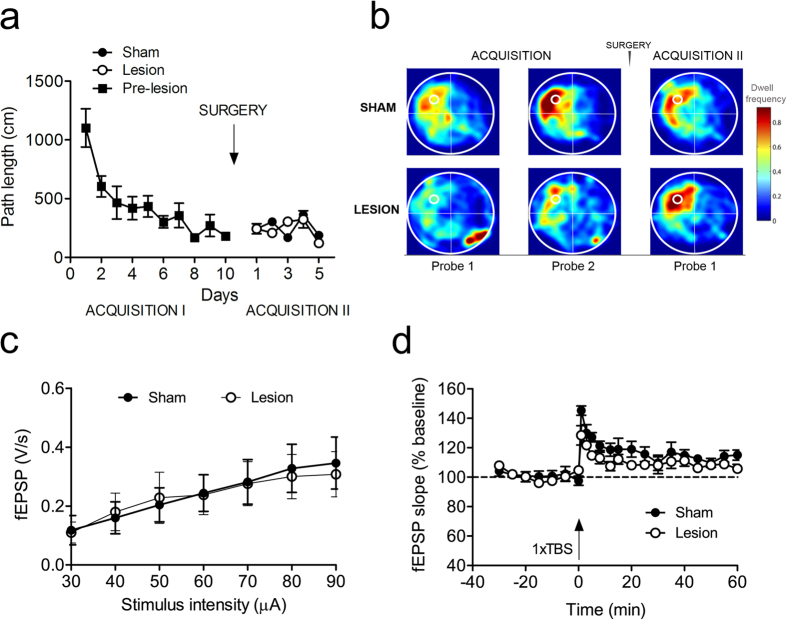
mPFC damage does not affect recall of learned spatial information, but does impair hippocampal LTP. (**a**) QA-injected mice perform equally to sham during 5 days of extended training in the MWM. Platform location is kept in the same position before and after the surgery (time of the surgery indicated by arrow). (**b**) Heat maps illustrate near identical performance in the two groups in time spent searching the target quadrant during probe tests, (**c**) No changes in basal synaptic transmission (as shown by the I/O curve) between treatment groups. (**d**) QA-injected mice expressed only short-lasting potentiation of about 10 min duration, whereas sham mice developed short-term potentiation that returned to baseline after about 35 min.

To examine whether the effect of mPFC lesions was specific to reversal learning performance, we trained another batch of mice for 10 consecutive days in the MWM, injected QA as before, and then simply continued with the same platform configuration (EXP3). In contrast to the reversal learning condition, mPFC lesions did not affect performance, indicating that mPFC is not required to recall learned spatial information (Fig. 6a). Likewise, the probe trial conducted on day 16 after 15 days of training did not reveal any difference between the two groups as shown in the heat maps constructed using summed dwell time for every animal (Fig. 6b). Electrophysiological CA1 recordings on slices from lesioned and sham animals did not detect differences in basal synaptic transmission (I/O curve; Fig. 6c), whereas single TBS-evoked potentiation was significantly stronger in sham than in lesioned animals. This resulted in a significant group difference during the first 30 min after TBS stimulation (F_1,8_ = 12.2, p < 0.01, RM-ANOVA; Fig. 6d). Non-linear regression with a one-phase exponential decay yielded a significantly higher value for the plateau of potentiation in sham mice (114.3 ± 2.95%) as compared with lesioned animals (108.05 ± 2.2%; p < 0.05). When basic excitability and LTP were compared between reversal learning (Fig. 5a,b) and continued MWM training (Fig. 6c,d), the reversal learning group displayed marginally lower excitability (F_1,17_ = 3.2, p = 0.089) and declined maintenance of potentiation (F_1,14_ = 6.1, p = 0.027).

## Discussion

In the present report, we investigated the functional effects of excitotoxic QA injection into mPFC, and also assessed the long-assumed validity of this procedure as a chemical model of early neurodegeneration. Reduced synaptic density in PFC is the strongest pathological correlate of cognitive decline and severity of dementia^45^. Deficits in executive functions and cognitive flexibility might in fact be early indicators of degenerative PFC pathology^46^,^47^, and executive dysfunctions were consequently reported in mouse models of frontotemporal dementia^48^. Interestingly, QA targets axo-dendritic NMDA receptors, which ensures that mPFC neurons, and not passing fibers, are affected. Such experimentally induced focal brain lesions have been instrumental to our present understanding of the complex anatomical and functional organization of brain systems, including PFC-HC interactions and their relation to cognitive performance^26^,^49^,^50^,^51^.

The endogenous tryptophan metabolite, QA, has similar potency at the NMDAR to glutamate, but its excitotoxic actions are stronger, because QA remains much longer in the synaptic cleft, due to less-efficient uptake^52^. The excitotoxic effect of QA is influenced by NMDAR subunit composition^52^,^53^ and modulated by metabotropic glutamate receptors^54^. QA shares with NMDA its property to damage preferentially pyramidal neurons, while sparing other neuron types^55^. Notably, QA has been shown to produce oxidative damage, independent of its activity at the NMDAR, by forming complexes with Fe^2+^ that mediate the generation of reactive oxygen species (ROS)^56^,^57^. This effect might be further enhanced by inhibitory effects of QA on antioxidant enzymes. Other neurotoxic actions of QA include hyperphosphorylation of tau cytoskeleton proteins^58^,^59^, induction of proinflammatory cytokines, mitochondrial inhibition leading to energetic deficits, activation of caspases, and enhancement of cytochrome c release^2^,^52^. By and large, the multiple actions of QA reproduce many neuropathological processes that render this endogenous excitotoxin a valuable tool to study the mechanisms underlying neurodegeneration^2^,^52^,^60^.

Results of EXP1 showed that QA injection into mPFC acutely increased basal excitability and enhanced HC synaptic plasticity (LTP). These synaptic changes and lesions most likely involved NMDAR-mediated excitotoxicity following excessive Ca^2+^ influx. Secondarily, QA induces oxidative stress and glial cell activation that also occur during neurodegeneration^2^. Notably, our results showed that QA injections into mPFC PL/IL regions affected connected regions such as HC differently depending on the time after injection (i.e., initial excitation, followed by degeneration). Indeed, 24 h after injection, we found a marked increase in basic HC excitability and LTP strengthening, whereas two weeks later, we observed reduced HC excitability and severely impaired LTP. Prefrontal QA injection apparently affected crucial prefrontal input to HC, which has been shown to originate from anterior cingulate (AC)-HC pathway^61^ and indirectly from *nucleus reuniens*^24^,^25^. The eventual changes in HC synaptic transmission likely resulted from a time-dependent, QA-induced pattern of increased and decreased PFC input, which may actually be similar to the pathological processes occurring during the different stages of neurodegeneration. However, the processes that underlie the effect of prefrontal QA injection on HC circuitry and signaling cascades, as well as their putative relationship to pathological neurodegeneration definitely remain to be further investigated.

The changes in hippocampal LTP, both acutely and more delayed, indicate that the glutamatergic PFC-HC pathway was affected by the injection of this glutamate analogue into mPFC. Previous reports that focused on the connection between PFC and HC implemented these interconnected regions in different cognitive processes^19^,^29^,^62^. Chudasama *et al.*^29^ specifically argued that connections between PFC and HC are crucial for behavioral flexibility and response inhibition. MWM learning in general depends on HC-mediated spatial functions^63^,^64^. However, QA-induced mPFC lesions did not affect the ability of mice to execute the MWM as such, but rather impaired their ability to update their spatial map and adjust their spatial-cognitive strategies (during MWM reversal). Mice with mPFC lesions were still able to locate the platform when it remained in the same position, but faced difficulties to acquire the new position when the location was changed. This first of all confirms the value of MWM reversal procedures to detect subtle cognitive changes such as those following mPFC lesions^65^,^66^, but also shows that mPFC is specifically involved in tasks that require the adjustment of previously learned contingencies and cognitively demanding search strategies. Defects in the deployment of spatial strategies have been described in other brain disease models as well^67^,^68^,^69^, whereas rigidity in the deployment of efficient strategies to locate the hidden platform appear to underlie the presently observed impairments. Mice with mPFC lesions were unable to adjust their spatial maps and search strategies, and rather relied on a mixture of different non-spatial and repetitive strategies.

We used rsfMRI as a non-invasive methodology to examine the effects of mPFC lesions on cortical FC. Notably, we found that QA injections into mPFC actually increased FC strength between various cortical regions, which might in fact mimic the early, excitatory stages of Alzheimer-like neuropathology^70^. One of the advantages of the rsfMRI methodology is its ability to detect functional alterations that occur much earlier than structural changes^37^,^71^,^72^. The reversal learning defects and spatial-cognitive rigidity, which we observed in our mPFC-lesioned mice, coincided with widespread changes in cortical connectivity. This supports the notion that MWM reversal depends on functional brain circuitry that includes PFC and associated cortical structures. PFC microcircuitry has been shown to regulate incoming inputs from other brain regions by providing feedback signals that are required to perform a particular task^73^. The pattern of deficits following PFC damage is consistent with a loss of the ability to maintain task-relevant information and use behavior-guiding rules^49^,^74^. PFC activity must be maintained during a specific task as long as the rule to perform the task is required, and PFC neurons indeed appear to remain active after learning new associations^73^.

Increased and probably dysfunctional cortical connectivity is likely to have resulted from the lack of attenuated control that is physiologically exerted by the PFC. Our results indicate that increased cortical connectivity does not correspond with better or more adaptive cognitive performance. Rather on the contrary, increased cortical connectivity coincided with a reversal defect in our QA-injected mice. Most likely, hyperconnectivity induced by QA injection is the result of the temporary excitotoxic hyperactivation that spreads to connected regions and induces LTP-like increases in connectivity that ultimately leads to neuronal loss. Interestingly, the observed hyperconnectivity was accompanied by deficits in reversal learning, whereas the recall of previously learned information as well as the deployment of acquired spatial-cognitive strategies were unaffected. Connectivity between HC, mPFC and associated cortical areas appears to be important when animals need to adjust their behavior by learning new contingencies and repress previously established ones.

We have damaged mPFC including PL and IL, and our lesioned animals may therefore have experienced difficulties in various mPFC-controlled functions such as (1) inhibition of previously learned association, (2) shifting to a new strategy, and (3) using spatial information to execute the new strategy. Damage to mPFC impaired reversal learning performance in accordance to the putative functions of this brain area in behavioral flexibility^35^, and consistent effects of mPFC excitotoxic lesions^27^,^75^,^76^,^77^.

Finally, we also examined synaptic plasticity in hippocampal CA1 region of mice that had received QA injection several days previously. Plasticity readouts such as LTP recordings are often used to assess synaptic functionality and the ability of synapses to achieve long-term activity-dependent changes^78^. The enhanced LTP observed 24 hours after QA injection has been a striking finding which points to both, (i) the potency of excitatory signals that are transmitted after QA injections in changing synaptic functionality in pathways and regions that are connected to the injected region and (ii) the sensitivity of LTP to detect such signals. The electrophysiological recordings performed about 2 weeks after QA lesions revealed a significant reduction of LTP in the lesioned mice, which coincided with their reversal defect. It was probable to expect that changes in input, originating from a damaged mPFC, would affect HC physiology, given the reciprocal anatomical pathway between mPFC and HC, and the (albeit poorly understood) electrophysiological connection between these regions^22^. However, while QA-induced mPFC lesions led to an initial enhancement of CA1-LTP, probably through an excitotoxic hyperexcitability, the decreased input from the damaged mPFC at later stages resulted apparently in a down-regulation of synaptic transmission and LTP, possibly by mechanisms of homeostatic synaptic plasticity^79^,^80^. The deficit in synaptic functioning provides a mechanistic rationale for the impaired reversal learning of the lesion group. The different magnitude of the LTP deficits in lesioned animals observed in EXP2 vs. EXP3 indicates that natural stimuli such as a reversal learning task impose stronger functional demands on the synaptic circuitry that lead to, and become overt as, more pronounced deficits in behavioural or electrophysiological read-outs. It has been reported that exposure to environmental novelty leads to synaptic resetting in CA1^81^. Hence, the downscaling of synaptic transmission in response to the functional silencing of mPFC by QA and the functional demands of the synaptic resetting during reversal learning are likely to have caused together the behavioral and LTP-deficits observed in our study.

In conclusion, these observations demonstrate the central functional importance of rodent mPFC as well as the validity of QA-induced mPFC damage as a preclinical rodent model of the early stages of neurodegeneration.

## Additional Information

**How to cite this article**: Hernandez, A. L. *et al.* Quinolinic acid injection in mouse medial prefrontal cortex affects reversal learning abilities, cortical connectivity and hippocampal synaptic plasticity. *Sci. Rep.* **6**, 36489; doi: 10.1038/srep36489 (2016).

**Publisher’s note**: Springer Nature remains neutral with regard to jurisdictional claims in published maps and institutional affiliations.

## Figures and Tables

**Table 1:** Numbers of mice used in each of the three experiments.

Experiment and Methodology		Lesion	Sham
EXP1	Hippocampal LTP	6	7
EXP2	MWM Reversal	13	10
	rsfMRI	12	10
	Hippocampal LTP	11	9
EXP 3	MWM Extended Training	6	5
	Hippocampal LTP	6	5

**Table 2:** Classification of search strategies during MWM acquisition and reversal.

Main Strategy	Subclass	Description
Spatial	Spatial direct	Swimming directly to the platform
	Spatial indirect	Swimming with just one explorative loop towards the platform
	Focal correct	Searching in the correct quadrant
Non-spatial	Scanning	Searching in the center of the pool
	Random	Failure to show a preference for any part of the pool
	Focal incorrect	Searching in the wrong quadrant
Repetitive	Chaining	Circular swimming in the target annulus area
	Peripheral looping	Wall hugging (thigmotaxis)
	Circling	Swimming in tight circles
Perseveration		Searching the platform in the previous rewarded quadrant
